# Reliability and accuracy of interview data in non-smoking female lung cancer case-control study

**DOI:** 10.1186/1756-9966-27-43

**Published:** 2008-09-24

**Authors:** Desheng Huang, Peng Guan, Hailong Shi, Qincheng He, Baosen Zhou

**Affiliations:** 1 Department of Epidemiology, School of Public Health, China Medical University, Shenyang 110001, PR China; 2 Key Laboratory of Cancer Etiology and Intervention, University of Liaoning Province, PR China

## Abstract

**Background:**

Valid interview data is critical to the final results of the study. The purpose of this study was to investigate the reliability of epidemiological data obtained in non-smoking female lung cancer case-control study in China.

**Methods:**

Fifty-six pairs of cases and controls, 10% percent of all the collected subjects were re-interviewed by three interviewers who underwent identical standardized training. A limited number of questions included in the original survey were asked again, the responses from the re-interview were compared with the original interview. Kappa was calculated by negative rates of agreement, positive rates of agreement and total rates of agreement to the accordance degree between the two interviews.

**Results:**

The Kappa values were all more than 0.5 in all the studied indexes. The Kappa values descended from 0.92 in family history of cancer to 0.56 in oral contraception use. Errors in collecting and classifying data did occur, and were especially common for complicated clinical events, such as a drug exposure occurring many years before.

**Conclusion:**

We identified four sources of this variability, three in collecting the data, and one in coding. As a result of these findings, strategies are proposed for improving the quality of interview data obtained in epidemiological research. Before finding a good solution, the strategy of data collecting and coding should be simple and easy to inspect.

## Background

Lung cancer is one of the leading causes of cancer death worldwide and in China [1-6]. All those studies showed that cigarette smoking [7-9] was the primary risk factor for female lung cancer, which accounted for only 25% of all female cell types and 10% of female adenocarcinoma in Shanghai [10], 37% of all female cell types and 14% of female adenocarcinoma in Shenyang [11]. These statistics pointed to the necessity of investigating and understanding the etiology of lung cancer in general, and urgency for identifying factors affecting the risk for female lung cancer. To explain the reasons of the high incidence of Chinese female lung cancer, we investigated risk factors for non-smoking female lung cancer by using a case-control study design.

The interview data of this study were obtained through questionnaires. Previous studies have showed that some medical information could be remembered more accurately than others [12-14]. For example, people could remember details of illnesses requiring hospitalization better than illnesses treated in the physician's office. The patient's medical knowledge and anxiety level might determine the recall of the information [15]. However some interviewers of varying training and ability are always aware of hypothesized risk factors under investigation.

Valid interview data is critical to the final results of the study. We repeated the interview or medical record extraction at a later date to examine the reliability of interview data, identify sources of variability and provide solutions to minimize variation in data collection. The acceptability of the interview method and the adequacy of the management of the case-control study were also explored in this study.

## Methods

### Subjects

Five hundred and sixty newly diagnosed primary lung cancer cases in females, aged 35–74, defined according to the 3-digit rubric of ICD-9, were recruited in 18 hospitals serving the city of Shenyang from June, 2000 to June, 2004. Each case enrolled in this study was newly diagnosed with lung cancer based on reviews of relevant medical records, chest X-ray and CT films. About 67% cases had histological or cytological slides confirmed by a panel of chest physicians and pathologists. All the cases were nonsmoking females (defined as those who had not smoked more than 100 cigarettes or used other tobacco products for more than 6 months in their lifetime). Cancer-free controls were 1:1 matched by the same age group (± 2 years old) and randomly selected from the same administrative district or the same hospital during the same period as the cases. Controls had no previous or present history of malignant diseases including lung cancer. Controls that were recruited in hospitals suffered mainly from bronchitis, fibrosis and chronic obstructive pulmonary disease.

### Interviews and Re-Interviews

After detailed explanation of the study, all selected participants signed an informed consent form and agreed to be interviewed at their convenience. Three interviewers who underwent identical standardized training interviewed all of the cases and controls. Interviews, were carried out by direct conversation and medical record extraction, lasted 20–30 minutes and followed a structured questionnaire format. The questionnaire used in the first interview consisted of 6 different parts as follows: general personal information; living environmental and household habits; the situation of ETS (Environmental Tobacco Smoke) exposure; dietary habits; social economic status information; medical record (only for lung cancer cases and controls collected in hospitals). After completing the interview, the information was checked by a supervisor, coded and entered into computers for analysis.

Fifty-six pairs of cases and controls, 10% percent of all the collected subjects were randomly selected and re-contacted to verify the initial interview by asking a limited number of questions included in the original survey and re-extracting the medical records. The random sampling process was performed monthly as follows: after completing the first-round interview, each case and control was assigned a unique identification number. The supervisor checked the completed questionnaires and relevant medical information monthly, and concurrently assigned each pair of cases and controls a random number by a random-number generator. These random numbers were sorted in ascending order, then the top 10% of the random numbers were selected and the corresponding pairs of cases and controls were selected for re-interview. The repeated questions that needed to re-asked included certain features that were of potential importance to the analysis of the risk factors of lung cancer, such as family cancer history, disease history and cooking styles. All the repeated interviews were conducted within one month after the first interview. The three technicians who underwent the same identical standardized training with the above mentioned three interviewers carried out the re-interview. Each technician did not know the results of the first interview, and was kept unaware of the specific hypothesis under investigation. During the re-interview process, they were asked only to collect the following 5 aspects of information: history of illness, family history of cancer, oral contraceptive use, location of kitchen during childhood and history of passive smoking. The simple questionnaire adopted in the re-interview process was extracted from the structured questionnaire that was used in the first interview. The three technicians also collected any comments on the first interview from the participants during the re-interview process.

### Identifying Source of Variation

This study focused not only on measuring the amount of variation in collecting data from an interview, but also on identifying the reasons of the variation for better management of the case-control study. After completing the interviews, detailed analyses of disagreement were conducted and the disagreement were classified into four categories. Two of them could be attributed to subject disagreement and incomplete information. Because sometimes people provided information that contradicted data in the other interview and only provided partial information. Another source of variation was interviewer misinterpretation. This occurred when the interviewer incorrectly interpreted the information provided by the subject. The last source of variation was from coding misinterpretation. This occurred when the data coder interpreted the data differently on the similar data from the two interviews.

### Statistical analysis

The extent of agreement between interviews was assessed using per cent observed agreement for specific responses (positive and negative), and for per cent overall agreement. We also calculated an unweighted kappa statistic to provide an estimate of chance corrected agreement. The kappa statistic is calculated by the formula κ = (p_o_-p_c_)/(1-p_c_), where p_o _is the observed proportion of agreement, and p_c _is the proportion of agreement expected by chance. Kappa has a range of values from -1 to +1. When the observed agreement is perfect, i.e., p_o _= 1, kappa will be +1. If the observed agreement equals the chance expected agreement, so that p_o _= p_c_, kappa will be 0. If the observed agreement is less than the chance expected agreement, i.e., p_o _< p_c_, kappa will become negative. With the recommendations of several research statisticians [16], the kappa value is interpreted as follows: the rating of agreement is poor when the index is < 0.4; the rating is fair when the index is 0.4–0.59; the rating is good when the index is 0.60–0.75; and the rating of agreement is excellent when the index value is > 0.75.

## Results

### General and demographic distribution for cases and controls

The demographic information for cases and controls is presented in Table 1. There was no difference in the distribution of age, marital status, and educational level between cases and controls. The economic status (based on income in 1995, five years prior to interview) in the cases was higher than that in the controls and the difference was statistically significant.

**Table 1:** Characteristics of female lung cancer cases and controls

Variable	Cases	Controls	P value
Number	56	56	
Age(years)	53.4 ± 10.2	52.6 ± 9.6	0.52
Income(Monthly RMB yuan per person per family in 1995)	334.9	311.5	0.03
Level of Education			
None	13	7	
Primary school	14	21	
Junior school	13	17	
Senior school and high	16	11	0.20

P-value from t-test for continuous variables; χ^2 ^test for category variables.

### Interview variability

There was no difference in the extent of agreement among cases and controls. Table 2 shows the interview variability of five selected questions. For each item, we presented the total number of disagreement, the observed agreement and the kappa value. The observed agreement ranged from 88.39% for history of illness and location of kitchen during childhood to 99.11% for history of passive smoking. The Kappa value descended from 0.92 in family history of cancer to 0.56 in oral contraception use. The kappa statistic suggested fair, good or excellent agreement for all five variables.

**Table 2:** Kappa value for selected questions

	Total number of disagreement	Observed agreement (%)	Kappa
History of illness	13	88.39	0.75
Family history of cancer	3	97.32	0.92
Oral contraceptive use	12	89.29	0.56
Location of kitchen during Childhood	12	89.29	0.78
History of passive smoking	1	99.11	0.92

### Sources of disagreement

Classified sources of disagreement between interviews for five variables are presented in Table 3. Between the two interviews, there were a total of 41 disagreement. Disagreement was more frequent for oral contraceptive use, history of illness and location of kitchen during childhood than for family history of cancer. About 63% of the disagreement occurred because of conflicting patient reports, and 24% due to incomplete information.

**Table 3:** Sources of disagreement for interview comparison

	Feature					
		
Source of disagreement	History of illness	Family history of cancer	Oral contraceptive use	Location of kitchen during Childhood	History of passive smoking	Totals
1. subject disagreement	9	1	9	7	0	26
2. incomplete information	2	2	1	4	1	10
3. Interviewer misinterpretation	1	0	0	1	0	2
4. Coding misinterpretation	1	0	2	0	0	3
Totals	13	3	12	12	1	41

## Discussion

Questionnaires were widely used as a basic instrument for data collecting. Face to face interview with a structured questionnaire and hospital discharge abstract were adopted in our lung cancer case-control study of non-smoking females. To control and guarantee the quality of the collected data, we re-interviewed 10% of the subjects on limited questions, and compared the results with those of the original interviews.

The results of this study suggested that subjects could recall and report much clinical information during interviews for epidemiological studies. For some variables, such as family history of cancer and history of passive smoking, agreement between the two interviews was excellent, with both kappa values were more than 0.75. However for oral contraceptive use, the level of agreement for specific oral contraceptive brand, dose, total duration of use was low, which was of part concordance with the results of other previous studies [17-19]. Rosenberg MJ and etc. found that agreement was better for cases than for controls, when they compared the histories of oral contraceptive (OC) use provided by women participating in a study of hepatocellular adenoma (HCA) with records obtained from their physicians[18]. However, there was no difference in the extent of agreement among cases and controls in the present study. Those studies suggested that in studies examining the effects of individual substances or doses, researchers should try to obtain data from medical records, and user reports about previous OC use might be less useful and more suspect[17,19]. However in China, at present time we can not get the accurate data of oral contraceptive pharmacy records. Sometimes personal recall is the only way to gather this kind of information.

With the development of health promotion activities, such as health education, advertisements of public benefit etc., more and more people pay increasing attention to cancer and related risk factors. They have a clear understanding of the harmful effects of passive smoking. However, only a few people are aware of the exposure to cooking oil smoke and oral contraceptive use. The degree of awareness of the events in daily life is one of the determinants of the recall of information.

During the second interview, when asking the attitude towards the first interview, subjects were willing to support and participate in research but wanted to be consulted in detail on the use of information from their medical records. They were also concerned about secondary uses of their data, particularly for marketing and insurance purposes. These challenges call for new approaches to consent, taking the varying needs of the subjects and the evolving uses of personal information into account. So more detailed explanation of the study objectives and better collaboration with the doctors in charge and social workers in the community should be strengthened in the future to eliminate misgivings. The doctors and the social workers are critical in informing and motivating the target population to participate the study.

In our study, data coding was after data collection to save the interviewer's time and effort. But coding misinterpretation occurred. More communication between the coding technicians and the interviewer would avoid the coding misinterpretation. Detailed, explicit coding manuals and questionnaire explanations should be developed after these errors occurred. The results in our study showed that errors could occur in both data collection and data coding, better methods are needed to enhance the quality of the information.

## Conclusion

We identified four sources of this variability, three in collecting the data, and one in coding. As a result of these findings, strategies are proposed for improving the quality of interview data obtained in epidemiological research. Before finding a good solution, the strategy of data collecting and coding should be simple and easy to inspect.

## Competing interests

The authors declare that they have no competing interests.

## Authors' contributions

DH conceived the study and drafted the manuscript. DH, PG, HS and BZ managed and analyzed the data. QH attracted funding and managed the whole case-control study. All authors contributed to the writing of the final version of this paper.
